# 牙龈卟啉单胞菌在肺癌中的相关研究与进展

**DOI:** 10.3779/j.issn.1009-3419.2024.101.28

**Published:** 2024-10-20

**Authors:** Yuanyuan YIN, Jie ZHANG, Qiang GUO, Cheng SHEN

**Affiliations:** ^1^610041 成都，四川大学华西医院胸外科; ^1^Department of Thoracic Surgery, West China Hospital, West China Hospital of Stomatology, Sichuan University, Chengdu 610041, China; ^2^610041 成都，四川大学华西护理学院; ^2^West China School of Nursing, West China Hospital of Stomatology, Sichuan University, Chengdu 610041, China; ^3^610041 成都，四川大学，华西口腔医院，口腔疾病防治全国重点实验室; ^3^State Key Laboratory of Oral Diseases, West China Hospital of Stomatology, Sichuan University, Chengdu 610041, China

**Keywords:** 牙龈卟啉单胞菌, 肺肿瘤, 相关性, Porphyromonas gingivalis, Lung neoplasms, Correlation

## Abstract

牙龈卟啉单胞菌（Porphyromonas gingivalis）是引发牙周炎的一种核心病原微生物，不仅与慢性牙周炎等口腔疾病密切相关，近年来被发现与癌症的发生、发展及预后均具有显著相关性。肺癌作为全球发病与死亡人数均为首位的恶性肿瘤，一直都是研究者关注的重点与热点。肺癌的发病原因复杂，涉及多种因素，包括吸烟、职业因素、空气污染、电离辐射、饮食与营养、遗传因素等。研究者们也开始关注到口腔微生物群与全身健康的关系，特别是与肺癌之间的联系。本文总结了牙龈卟啉单胞菌在肺癌中的相关研究最新进展，主要包括病因、致病机制等，并探讨了其作为肺癌潜在治疗靶点的可能性，旨在为肺癌的预防和治疗策略提供新的思路与方向。

## Abstract (en)

Porphyromonas gingivalis is a key pathogenic microorganism that triggers periodontitis. It is closely associated with oral diseases such as chronic periodontitis and recently found to have a significant correlation with the occurrence, progression, and prognosis of cancer. As the leading malignant tumor in terms of both incidence and mortality worldwide, lung cancer has always been a focus and hotspot of research. The causes of lung cancer are complex and involve multiple factors, including smoking, occupational factors, air pollution, ionizing radiation, diet and nutrition, genetic factors, etc. Researchers have also begun to pay attention to the relationship between oral microbiota and overall health, especially the link with lung cancer. The article summarizes the latest advancements in research on Porphyromonas gingivalis in lung cancer, primarily encompassing etiology and pathogenic mechanisms, and explores its potential as a therapeutic target for lung cancer, aiming to provide new insights and directions for lung cancer prevention and treatment strategies.

随着大众体检意识的增强和检测设备的更加精准化，越来越多的肺部结节被发现，同时，大多数肺部结节是早期肺癌的影像学表现。据全球肿瘤流行病学研究^[1]^报道，2022年肺癌新发病例近250万，死亡病例超180万，占全球癌症发病的12.4%和死亡的18.7%，是男性癌症首位死因，女性中则仅次于乳腺癌。早期肺癌的治疗方式主要以外科手术为主^[2]^，针对中晚期的肺癌患者，术前新辅助靶向治疗方式也逐渐成为重要的治疗手段^[3]^。肺癌的发病原因复杂，涉及多种因素，包括吸烟、职业因素、空气污染、电离辐射、饮食与营养、遗传因素以及其他因素等^[4,5]^。社会的进步让人们也开始关注口腔微生物群与全身健康的关系，特别是与肺癌等慢性疾病之间的联系。牙龈卟啉单胞菌（Porphyromonas gingivalis）是一种革兰氏阴性厌氧细菌，它在宿主体内发挥着复杂的作用，能够调控免疫反应，加速微生物牙菌斑的形成与增长。这种细菌能够扰乱宿主的生理稳态，导致生态失衡，进而可能引发包括慢性牙周炎在内的多种疾病，甚至与某些恶性肿瘤的发生存在关联^[6,7]^。牙龈卟啉单胞菌作为牙周病的主要致病菌之一^[8]^，其在肺癌中的作用逐渐受到重视。本文旨在深入探讨牙龈卟啉单胞菌与肺癌之间的相关研究及其进展。

## 1 牙龈卟啉单胞菌的概述

牙龈卟啉单胞菌是一种革兰氏阴性厌氧菌，是牙周病的主要病原体，其主要毒力因子包含菌毛、脂多糖（lipopolysaccharide, LPS）、牙龈蛋白酶、血凝素（hemagglutinin, HA）等，可通过释放外膜囊泡（outer membrane vesicles, OM）的形式将LPS和牙龈蛋白酶等有效物质输送至远离其原始位置的地方，从而显著扩大了它们在宿主体内的影响范围与深度^[9]^，这些毒力因子通过多种机制破坏牙周组织，进而促进牙周病的进展^[10]^。

### 1.1 菌毛

菌毛是细菌表面的黏附结构，能够帮助细菌牢固地附着在牙齿表面或牙周组织上，为后续的破坏活动提供基础。牙龈卟啉单胞菌具备两种菌毛形态：一种是长菌毛，另一种是小菌毛。这些菌毛对于细菌与宿主组织之间的相互作用至关重要。其中长菌毛由FimA基因编码，其产物菌毛蛋白存在6种变体，牙龈卟啉单胞菌的长菌毛被分类为FimA I-V型以及特殊的Ib型，而小菌毛的蛋白成分由mfa1基因所编码。尽管这两种菌毛蛋白都在牙龈卟啉单胞菌的黏附和入侵机制中发挥作用，但它们在导致疾病方面的具体贡献是有所差异的。研究^[11]^显示，当FimA基因发生缺陷时，细菌无法形成典型的长菌毛结构，进一步的研究^[12,13]^还指出，菌毛的致病特性主要与FimA基因的不同变体紧密相关，而与mfa1基因编码的小菌毛蛋白的型别关系不大。其中，FimA II和FimA IV具有更强的黏附性和侵袭性，与牙周病有关^[7]^。

### 1.2 脂多糖

牙龈卟啉单胞菌的LPS，作为其OM的重要外层结构成分，不仅关乎细菌的侵袭性，而且涉及到细胞活动的调控、营养自给以及炎症的加剧，是连接细菌病理活性与宿主免疫反应的重要桥梁。LPS被发现能够刺激并促进多种细胞类型的增殖与迁移活动，表明其具备增强细胞生长与活动的潜力^[14,15]^。此外，它还负责维护细菌自身的营养供应。LPS通过与Toll样受体1（Toll-like receptor 1, TRL1）和TLR4相互作用，促进分泌细胞因子如白介素6（interleukin 6, IL-6）、IL-8和肿瘤坏死因子-α（tumor necrosis factor-α, TNF-α），并产生炎症^[16]^。

### 1.3 牙龈蛋白酶

牙龈蛋白酶是一种半胱氨酸蛋白酶，能够降解宿主组织中的蛋白质，为细菌提供所需的氨基酸等营养素，同时参与调节宿主的免疫反应，引起炎症反应和组织损伤^[17]^。它可分为精氨酸（RgpA和RgpB）和赖氨酸（Kgp）特异性半胱氨酸蛋白酶，其中RgpA和Kgp蛋白质在结构上展现出相似性，它们都整合了两种关键的功能区域——一个是催化结构域，另一个是HA结构域。相比之下，RgpB蛋白质较为简单，它仅包含催化结构域，缺乏HA结构域，这可能意味着RgpB在功能上更加专注于催化反应，而较少涉及与细胞或分子的直接结合^[18]^。

### 1.4 HA

HA是牙龈卟啉单胞菌表面的一种蛋白质，具有与红细胞和其他宿主细胞表面受体结合的能力。牙龈卟啉单胞菌通过其HA和牙龈蛋白酶的协同作用，能够有效地黏附于宿主细胞表面、降解宿主组织中的蛋白质、逃避宿主的免疫反应，从而获取自身生长所需的营养素^[19]^。

## 2 牙龈卟啉单胞菌与肺癌的相关性

### 2.1 病因

牙龈卟啉单胞菌是成人慢性牙周炎的主要原因^[20]^，而牙周病患者的口腔卫生状况往往不佳，这可能导致口腔细菌直接或间接介导的牙周病与不同类型的癌症（包括肺癌）之间存在关联^[21]^。从解剖结构上来看，口腔与下呼吸道紧密相连，构成了一个连续的通道，口腔内的微生物有可能进入下呼吸道，进而影响肺部的微生物环境^[22]^。流行病学调查结果^[23,24]^显示，口腔卫生不足、牙龈出血、牙齿脱落等口腔健康参数与肺癌易感性增加之间存在很强的相关性。

### 2.2 致病机制

近年来，科学研究^[25]^揭示了牙周病与肺癌之间存在的复杂而深刻的联系，特别是牙龈卟啉单胞菌在这一关联中扮演了关键角色。牙周袋深度是衡量牙周病严重程度的重要指标，它与肺癌发展有显著相关性^[26]^。Mai等^[27]^研究指出，牙周探诊深度受牙龈炎症和退缩影响，而牙槽嵴丧失高度则更能准确反映牙周长期受龈下细菌感染及宿主免疫反应的变化。绝经后妇女中，牙槽嵴丧失高度与肺癌风险增加有关。牙周病与多种微生物混合感染相关，特别是“红色复合体”如牙龈卟啉单胞菌等^[28]^。由于口腔与呼吸道相通，口腔细菌可能吸入肺部并定植，牙菌斑成为呼吸道致病菌的潜在来源^[29]^。已有研究^[30]^证明，牙龈卟啉单胞菌能够触发TLR2和TLR4的活化，并促使单核细胞释放IL-6、IL-8等炎性细胞因子，通过降低牙龈卟啉单胞菌的活性或抑制其相关信号通路，可能与肺癌的发生及发展有相关性。同时，肖燕等^[31]^研究发现牙龈卟啉单胞菌感染肺部上皮细胞后，能够引起肺部的炎症反应，并促进促凋亡因子Bax、Fas、FasL的表达，同时抑制促增殖因子Bcl-2、转化生长因子-β（transforming growth factor-β, TGF-β）的表达，从而导致肺炎的发生，造成肺部的损伤，进一步在肺部炎症与肺癌之间引起相互作用。这一发现揭示了牙周致病菌感染造成肺癌的机制，一是通过抑制肺部细胞的增殖与修复能力，二是加速肺部感染细胞的凋亡过程从而促进肺癌的发生。这两方面的作用共同导致了肺癌的发展。

牙龈卟啉单胞菌作为牙周病的一种重要致病菌，通过引发持续的炎症反应、干扰宿主免疫反应、产生基因毒性物质等多种机制，可能加速肺癌细胞的增殖、迁移和侵袭，促进肿瘤的恶性进展^[32]^，Zeng等^[33]^进行了一项meta分析，综合考察了5项队列研究，共涉及321,420名参与者。他们发现牙周病与肺癌的风险存在显著关联，风险比（hazard ratio, HR）为1.24，95%置信区间（confidence interval, CI）为1.13-1.36，异质性指数（I²）为30%。此外，这种相关性在女性人群中同样显著。这一结论不仅加深了我们对牙周病与全身健康之间关系的理解，也为肺癌的预防和治疗提供了新的视角和思路。另外，在2021年进行的一项研究^[34]^中，研究团队在需要对患者进行插管和机械通气的临床案例中，成功地从患者的声门下灌洗液样本中检测到了牙龈卟啉单胞菌的存在。这一发现进一步证实了口腔微生物，特别是牙龈卟啉单胞菌，在呼吸系统健康中的潜在影响，尤其是在患者接受特定医疗干预（如插管和机械通气）时，其口腔微生物群可能通过呼吸道进入肺部，从而对肺部健康产生影响。刘怡文等^[35]^采用免疫组织化学技术探究牙龈卟啉单胞菌感染情况，发现该菌感染与吸烟、饮酒、淋巴结转移及肺癌临床分期均有明显关联。这一发现提示，长期吸烟与过量饮酒可能严重扰乱口腔微生态平衡，为牙龈卟啉单胞菌等病原体提供了更有利的生存环境。另外，在微生物种类方面，一项前瞻性研究^[36]^揭示橙色复合细菌抗体与肺癌之间存在明确的正相关关系（HR=1.15, 95%CI: 1.02-1.29）。在男性群体中，这种关联更为紧密（HR=1.27, 95%CI: 1.08-1.49）。这一发现主要归因于中间假单胞菌和黑假单胞菌的抗体水平上升（HR=1.15, 95%CI: 1.04-1.26）。此外，尽管样本规模较小（n=40），研究仍提示牙龈卟啉单胞菌的细菌数量与肺癌之间存在正相关趋势。值得注意的是，中间假单胞菌和牙龈卟啉单胞菌的细菌数量与其抗体水平呈正相关。有确凿的证据^[37]^表明，个体罹患肺癌的风险与其血清中针对牙龈卟啉单胞菌的免疫球蛋白G（immunoglobulin G, IgG）抗体水平呈正相关。这一发现揭示了免疫系统对牙龈卟啉单胞菌的反应可能作为预测肺癌风险的一个免疫标志物，进一步强调了口腔健康与肺癌之间复杂的生物学联系。Yan等^[38]^的研究发现，与健康对照组相比，肺癌患者唾液中的嗜氧化细胞数量和细孔菌含量明显更高。值得注意的是，在未吸烟的女性肺癌患者中，唾液微生物群落的平衡被打破，表现为群落多样性和丰富度显著降低，提示牙科护理在预防肺癌方面可能具有潜在的重要性，改善口腔健康状况或许能够有助于减少肺癌的发病概率。未来的研究应进一步探索这些细菌在肺癌发病过程中的具体作用机制，并评估相关预防措施的实际效果。

### 2.3 检测方法

目前，临床上并没有直接针对牙龈卟啉单胞菌在肺癌中的特异性诊断方法。然而，可以通过一系列的临床手段来间接评估牙龈卟啉单胞菌的感染情况以及其与肺癌的相关性。

#### 2.3.1 口腔检查与细菌培养

通过专业的口腔检查，观察牙龈的健康状况，包括牙龈红肿、出血、退缩等迹象，这些都是牙龈卟啉单胞菌感染的可能表现。同时也可以从患者的口腔样本（如牙菌斑、唾液等）中分离并培养牙龈卟啉单胞菌，以确认其是否存在和感染程度。

#### 2.3.2 血清学检测

通过检测患者血清中针对牙龈卟啉单胞菌的特异性抗体水平，可以间接评估患者的感染状况。然而，这种方法可能受到多种因素的影响，如个体差异、感染时间等，因此其准确性可能有限。

#### 2.3.3 影像学检查

虽然影像学检查无法直接检测牙龈卟啉单胞菌的感染，但它们可以显示肺部病变的情况，为医生提供评估肺癌病情的依据。同时，结合患者的口腔健康状况和临床表现，医生可以综合考虑牙龈卟啉单胞菌感染与肺癌的相关性。

#### 2.3.4 组织病理学检查

对于疑似肺癌的患者，可以通过手术或穿刺等方法获取肺组织样本进行病理学检查。在检查过程中，可以观察肺组织中是否存在牙龈卟啉单胞菌的感染迹象，如菌斑、炎症反应等。然而，这种方法需要专业的病理学技术和设备，且为有创检查，因此其应用受到一定限制。

#### 2.3.5 分子生物学检测

随着分子生物学技术的发展，可以通过检测患者口腔或肺组织样本中的牙龈卟啉单胞菌DNA或RNA来确认其感染情况。这种方法具有较高的敏感性和特异性，但同样需要专业的实验室技术和设备。

既往的研究中，患者的口腔、呼吸道及血清样本中，牙龈卟啉单胞菌的检出率明显高于非肺癌人群。例如，牙龈卟啉单胞菌可能通过引发持续的炎症反应、干扰宿主免疫反应、产生基因毒性物质等方式，促进肺癌细胞的增殖、迁移和侵袭^[39]^。牙龈卟啉单胞菌感染与吸烟、饮酒等肺癌风险因素密切相关^[32]^。这些不良生活习惯可能加剧口腔菌群失调，为牙龈卟啉单胞菌等有害菌的增殖提供有利条件。同时，口腔卫生状况不佳也是牙龈卟啉单胞菌感染的重要风险因素之一^[33]^。因此，维护良好的口腔卫生对于预防肺癌等全身性疾病具有重要意义。另有研究^[40]^发现，特定口腔细菌（牙龈卟啉单胞菌）与口腔鳞状细胞癌及肺癌中程序性细胞死亡配体1（programmed cell death ligand 1, PD-L1）上调有关，这为口腔微生物与全身疾病关联及肿瘤免疫疗法提供了新线索。有研究^[41]^显示，即便考虑吸烟因素，牙周病严重性仍与肺癌风险增加密切相关。未来研究可扩大至更多人群。由此可见，虽然目前并没有直接针对牙龈卟啉单胞菌在肺癌中的特异性诊断方法，但可以通过一系列的临床手段来间接评估其感染情况以及与肺癌的相关性。未来随着科学技术的不断进步和研究的深入，相信会有更加准确和便捷的诊断方法出现。

### 2.4 诊断

科技进步促进了低剂量螺旋计算机断层扫描（computed tomography, CT）的广泛应用，并显著提高了肺癌的早期发现率^[28]^。口腔微生物检测虽然不是肺癌的常规诊断手段，但是病原微生物的长期定植与肿瘤发生发展之间存在重要的病理学关联，刘怡文等^[35]^选择肺癌患者手术切除的癌组织及相应癌旁肺组织石蜡包埋标本为研究对象，采用免疫组织化学方法检测其牙龈卟啉单胞菌感染情况，并分析牙龈卟啉单胞菌感染与肺癌患者临床病理特征的相关性；同时，采用Kaplan-Meier生存分析法绘制生存曲线，分析牙龈卟啉单胞菌感染与生存时间之间的相关性，该研究结果显示，相较于肺腺癌组织，小细胞肺癌和肺鳞癌组织中牙龈卟啉单胞菌的感染阳性率更高，且这三种肺癌组织的免疫组化评分均明显高于癌旁肺组织。此外，肺癌患者的牙龈卟啉单胞菌感染与吸烟、饮酒习惯、淋巴结转移以及临床分期密切相关。值得注意的是，牙龈卟啉单胞菌感染阳性的肺癌患者5年生存率及中位生存时间均显著低于阴性患者。在对比3种肺癌病理类型时，发现肺鳞癌患者在牙龈卟啉单胞菌感染阴性组与阳性组之间的中位生存时间差异最为显著，超过了肺腺癌和小细胞肺癌患者。这些发现提示，长期吸烟和饮酒可能恶化口腔环境，使牙龈卟啉单胞菌更易感染并定植，从而加速肺癌的恶化进程。因此，有效清除牙龈卟啉单胞菌感染可能有助于延长肺癌患者，特别是肺鳞癌患者的生存期。由此说明，牙龈卟啉单胞菌等特定微生物的检测，可能有助于评估肺癌的风险或预后。进一步的研究发现，从肺癌患者的支气管液中分离出了牙龈卟啉单胞菌样菌株，这一发现为两者之间的微生物学联系提供了直接证据^[30]^。值得关注的是，牙龈卟啉单胞菌在肺癌组织中的定植比例明显高于癌旁肺组织，并且其感染状况与吸烟、饮酒习惯、淋巴结转移情况以及临床分期有紧密联系^[31]^。这一发现不仅强化了口腔微生物群在肺癌发生和发展中潜在作用的认识，还提示了不良生活习惯与口腔菌群失调可能共同促进肺癌的恶性进展。

## 3 展望

未来可以进一步深入研究牙龈卟啉单胞菌与肺癌之间的具体机制，包括其如何通过分子途径影响肺癌细胞的生物学行为，以及如何与宿主免疫系统相互作用等。这将有助于我们更全面地理解这一关系的本质，并为肺癌的预防和治疗提供新的思路和方法。基于现有研究成果，可以探索开发针对牙龈卟啉单胞菌的有效干预策略，如口腔清洁剂、疫苗或免疫疗法等。这些策略旨在减少口腔中牙龈卟啉单胞菌的数量或降低其致病性，从而降低肺癌的发病风险。牙龈卟啉单胞菌与肺癌之间的关系涉及多个学科领域，包括口腔医学、呼吸病学、肿瘤学、免疫学等。因此，需要加强跨学科合作，共同推进这一领域的研究进展。通过整合不同学科的优势资源和技术手段，可以更加全面地揭示这一关系的内在机制，并开发出更加有效的预防和治疗策略。未来的研究还可以进一步扩大样本量和研究范围，以验证现有研究成果的可靠性和普适性。同时，还可以探索不同人群、不同地域以及不同肺癌类型中牙龈卟啉单胞菌与肺癌之间的关系是否存在差异。这将有助于我们更全面地了解这一关系的复杂性和多样性。

## References

[1] Bray F, Laversanne M, Sung H, et al. Global cancer statistics 2022: GLOBOCAN estimates of incidence and mortality worldwide for 36 cancers in 185 countries. CA Cancer J Clin, 2024, 74(3): 229-263. doi: 10.3322/caac.21834 38572751

[2] National Health Commission Office. Guidelines for diagnosis and treatment of primary lung cancer (2022 edition). Xiehe Yixue Zazhi, 2022, 13(4): 549-570.

[3] Tsuboi M, Weder W, Escriu C, et al. Neoadjuvant osimertinib with/without chemotherapy versus chemotherapy alone for *EGFR* mutated resectable non-small-cell lung cancer: NeoADAURA. Future Oncol, 2021, 17(31): 4045-4055. doi: 10.2217/fon-2021-0549 34278827 PMC8530153

[4] Huang Y, Zhu M, Ji M, et al. Air pollution, genetic factors, and the risk of lung cancer: a prospective study in the UK biobank. Am J Respir Crit Care Med, 2021, 204: 817-825. doi: 10.1164/rccm.v205erratum2 34252012

[5] So R, Chen J, Mehta AJ, et al. Long-term exposure to air pollution and liver cancer incidence in six European cohorts. Int J Cancer, 2021, 149: 1887-1897. doi: 10.1002/ijc.33743 34278567

[6] Gasmi Benahmed A, Kumar Mujawdiya P, Noor S, et al. Porphy-romonas gingivalis in the development of periodontitis: impact on dysbiosis and inflammation. Arch Razi Inst, 2022, 77(5): 1539-1551. doi: 10.22092/ARI.2021.356596.1875 37123122 PMC10133641

[7] Singh S, Singh AK. *Porphyromonas gingivalis* in oral squamous cell carcinoma: a review. Microbes Infect, 2022, 24(3): 104925. doi: 10.1016/j.micinf.2021.104925 34883247

[8] Reyes L. Porphyromonas gingivalis. Trends Microbiol, 2021, 29(4): 376-377. doi: 10.1016/j.tim.2021.01.010 33546976

[9] Gui MJ, Dashper SG, Slakeski N, et al. Spheres of influence: *Porphyromonas gingivalis* outer membrane vesicles. Mol Oral Microbiol, 2016, 31(5): 365-378. doi: 10.1111/omi.12134 26466922

[10] Gabarrini G, Grasso S, van Winkelhoff AJ, et al. Gingimaps: protein localization in the oral pathogen *Porphyromonas gingivalis*. Microbiol Mol Biol Rev, 2020, 84(1): e00032-19. doi: 10.1128/MMBR.00032-19 31896547 PMC6941882

[11] Andrian E, Grenier D, Rouabhia M. *Porphyromonas gingivalis*-epithelial cell interactions in periodontitis. J Dent Res, 2006, 85(5): 392-403. doi: 10.1177/154405910608500502 16632751

[12] Hasegawa Y, Nagano K. *Porphyromonas gingivalis* FimA and Mfal fimbriae: current insights on localization, function, biogenesis, and genotype. Jpn Dent Sci Rev, 2021, 57: 190-200. doi: 10.1016/j.jdsr.2021.09.003 34691295 PMC8512630

[13] Amano A, Choi YH, Takeuchi H. Genotyping of *Porphyromonas gingivalis* in relationship to virulence. Methods Mol Biol, 2021, 2210: 53-59. doi: 10.1007/978-1-0716-0939-2_6 32815127

[14] Li X, Hu LL, He QM, et al. Effect of lipopolysaccharide from *Porphyromonas gingivalis* on the proliferation and migration of human umbilical artery smooth muscle cells in a coculture system. Zhonghua Kouqiang Yixue Zazhi, 2021, 56(6): 549-556. 34098670 10.3760/cma.j.cn112144-20210204-00062

[15] Utispan K, Pugdee K, Koontongkaew S. *Porphyromonas gingivalis* lipopolysaccharide-induced macrophages modulate proliferation and invasion of head and neck cancer cell lines. Biomed Pharmacother, 2018, 101: 988-995. doi: 10.1016/j.biopha.2018.03.033 29635909

[16] Mojtahedi H, Khannazer N, Mahmoud Hashemi S, et al. Effects of lipopolysaccharide from *Porphyromonas gingivalis* and *Escherichia coli* on gene expression levels of toll-like receptors and inflammatory cytokines in human dental pulp stem cells. Iran J Immunol, 2022, 19(3): 299-310. doi: 10.22034/iji.2022.92223.2136 36190383

[17] Xu W, Zhou W, Wang H, et al. Roles of *Porphyromonas gingivalis* and its virulence factors in periodontitis. Adv Protein Chem Stuct Biol, 2020, 120: 45-84. doi: 10.1016/bs.apcsb.2019.12.001 PMC8204362 32085888

[18] Liu J, Zeng JJ, Wang XX, et al. P 53 mediates lipopolysaccharide-induced inflammation in human gingival fibroblasts. J Periodontol, 2018, 89(9): 1142-1151. doi: 10.1002/JPER.18-0026 29964297

[19] Olsen I, Yilmaz Ö. Possible role of *Porphyromonas gingivalis* in orodigestive cancers. J Oral Micmbiol, 2019, 11(1): 1563410. doi: 10.1080/20002297 PMC6327928 30671195

[20] Hajishengallis G, Darveau RP, Curtis MA. The keystone-pathogen hypothesis. Nat Rev Microbiol, 2012, 10(10): 717-725. doi: 10.1038/nrmicro2873 22941505 PMC3498498

[21] Wang J, Yang X, Zou X, et al. Relationship between periodontal disease and lung cancer: A systematic review and *meta*-analysis. J Periodontal Res, 2020, 55(5): 581-593. doi: 10.1111/jre.12772 32583879

[22] Bassis CM, Erb-Downward JR, Dickson RP, et al. Analysis of the upper respiratory tract microbiotas as the source of the lung and gastric microbiotas in healthy individuals. mBio, 2015, 6: e00037. doi: 10.1128/mBio.00037-15 25736890 PMC4358017

[23] Kumar S. From focal sepsis to periodontal medicine: a century ofexploring the role of the oral microbiome in systemic disease. JPhysiol, 2017, 595(2): 465 -476. doi: 10.1113/JP272427 27426277 PMC5233655

[24] Scannapieco FA, Binkley CJ. Modest reduction in risk for ventilator-associated pneumonia in critically ill patients receiving mechanical ventilation following topical oral chlorhexidine. J Evid Based Dent Pract, 2012, 12(3 Suppl): 15-17. doi: 10.1016/S1532-3382(12)70004-0 23253825

[25] Chung M, York BR, Michaud DS. Oral health and cancer. Curr Oral Health Rep, 2019, 6(2): 130-137. doi: 10.1007/s40496-019-0213-7 31871854 PMC6927401

[26] Chrysanthakopoulos NA. Correlation between periodontaldisease indices and lung cancer in Greek adults: a case-control study. Exp Oncol, 2016, 38(1): 49-53. 27031720

[27] Mai XD, LaMonte MI, Hovey KM, et al. Periodontal disease severity and cancer risk in postmenopausal women: the Buffalo OsteoPerio Study. Cancer Causes Control, 2016, 27(2): 217-228. doi: 10.1007/s10552-015-0699-9 26661782 PMC4724219

[28] Mai XD, Genco RI, LaMonte MI, et al. Periodontal pathogens and risk of incident cancer in postmenopausal females: The Buffalo OsteoPerio study. J Periodontol, 2016, 87(3): 257-267. doi: 10.1902/jop.2015.150433 26513268 PMC4915107

[29] Wang GP. Defining funetional signatures of dysbiosis in periodontitis progression. Genome Med, 2015, 7(1): 1-3. doi: 10.1186/s13073-015-0165-z 25926890 PMC4414443

[30] Hutcherson JA, Bagatikar J, Nagano K, et al. Porphyromonas gingivalis RagB is a proinflammatory signal transducer and activator of transcription 4 agonist. Mol Oral Microbiol, 2015, 30(3): 242-252. doi: 10.1111/omi.12089 25418117 PMC4624316

[31] Xiao Y, Liu L, Shen KQ, et al. The effect of Porphyromonas gingivalis on the proliferation and apoptosis of lung epithelial cells. Guangdong Yixue, 2020, 41(11): 1145-1149.

[32] Teles FRF, Alawi F, Castilho RM, et al. Association or causation? Exploring the oral microbiome and cancer links. J Dent Res, 2020, 99(13): 1411-1424. doi: 10.1177/0022034520945242 32811287 PMC7684840

[33] Zeng XT, Xia LY, Zhang YG, et al. Periodontal disease and incident lung cancer risk: A meta-analysis of cohort studies. J Periodontol, 2016, 87: 1158-1164. doi: 10.1902/jop.2016.150597 27294431

[34] Morillo CM, Saraiva L, Romito GA, et al. Periodontopathogenic bacteria in subglottic samples from patients undergoing elective intubation for general anesthesia: A pilot study. J Periodontol, 2021, 92(8): e94-e102. doi: 10.1002/JPER.19-0570 33543507

[35] Liu YW, Yuan X, Sun W, et al. Clinical significance and prognostic value of Porphyromonas gingivalis infection in lung cancer. Jianan Daxue Xuebao (Ziran Kexue Yu Yixue Ban), 2020, 41(3): 260-267.

[36] Zhou B, Lu J, Beck JD, et al. Periodontal and other oral bacteria and risk of lung cancer in the Atherosclerosis Risk in Communities (ARIC) Study. Cancer Epidemiol Biomarkers Prev, 2023, 32(4): 505-515. doi: 10.1158/1055-9965.EPI-22-0601 35999656 PMC9947191

[37] Ampomah NK, Teles F, Martin LM, et al. Circulating IgG antibodies to periodontal bacteria and lung cancer risk in the CLUE cohorts. JNCI Cancer Spectr, 2023, 7(3): pkad029. doi: 10.1093/jncics/pkad029 37040077 PMC10152995

[38] Yan X, Yang M, Liu J, et al. Discovery and validation of potential bacterial biomarkers for lung cancer. Am J Cancer Res, 2015, 5(10): 3111-3122. 26693063 PMC4656734

[39] Baima G, Minoli M, Michaud DS, et al. Periodontitis and risk of cancer: Mechanistic evidence. Periodontol 2000, 2024, 96(1): 83-94. doi: 10.1111/prd.12540 PMC11579815 38102837

[40] Groeger S, Denter F, Lochnit G, et al. Porphyromonas gingivalis cell wall components induce programmed death ligand 1 (PD-L1) expression on human oral carcinoma cells by a receptor-interacting protein kinase 2 (RIP2)-dependent mechanism. Infect Immun, 2020, 88(5): e00051-20. doi: 10.1128/IAI.00051-20 32041789 PMC7171240

[41] Michaud DS, Lu J, Peacock-Villada AY, et al. Periodontal disease assessed using clinical dental measurements and cancer risk in the ARIC study. J Natl Cancer Inst, 2018, 110(8): 843-854. doi: 10.1093/jnci/djx278 29342298 PMC6093423

